# Monitoring youth-appealing advertising on social media for the e-cigarette brand Lost Mary

**DOI:** 10.18332/tpc/195631

**Published:** 2024-11-29

**Authors:** Michelle Jeong, Ollie Ganz, Eugene M. Talbot, Melanie LaVake, Olivia A. Wackowski, Patrick V. Barnwell, Scott I. Donaldson, Cristine D. Delnevo

**Affiliations:** 1 Rutgers Institute for Nicotine and Tobacco Studies, Rutgers Biomedical and Health Sciences, Rutgers University, New Brunswick, United States; 2 Department of Health Behavior, Society and Policy, Rutgers School of Public Health, Rutgers University, Piscataway, United States; 3 Division of General Internal Medicine, Rutgers Robert Wood Johnson Medical School, Rutgers University, New Brunswick, United States

**Keywords:** e-cigarettes, advertising, social media, youth appeal, Lost Mary

## Abstract

Lost Mary is a popular e-cigarette brand among youth in the United States. This study aimed to analyze Lost Mary’s branded social media activity and assess whether marketing efforts may have contributed to its youth appeal. We conducted a content analysis of 53 Lost Mary posts published on social media platforms from 2022 to 2023. More than a third of the posts included flavor descriptors, including fruit or ice, both popular flavors among youth. More than two-thirds of the posts used vibrant colors, and any human models included appeared to be young adults. Findings suggest that Lost Mary may target youth on social media platforms through the use of youth-appealing advertisement strategies (e.g. flavors). These findings highlight the need for increased monitoring of e-cigarette brand marketing on social media.

## INTRODUCTION

Lost Mary is an electronic cigarette (e-cigarette) brand (owned by iMiracle Shenzhen, the same company that owns ElfBar) with high youth appeal in the United States (US)^1^. Lost Mary was the subject of recent actions by the US Food and Drug Administration (FDA), including warning letters issued to retailers for selling this illegal, unauthorized product^2^. According to Nielsen Convenience Store sales data, Lost Mary first appeared in the US market in 2022 and showed rapid growth in product sales in 2023^3^. Its main mode of marketing is online, especially on social media – a concerning source of tobacco promotion worldwide that appeals to youth. Some social media platforms may restrict paid advertising^4^, but many tobacco and e-cigarette brands, including Lost Mary, have official accounts where they promote their products. Exposure to tobacco content on social media has been associated with youth use susceptibility^5,6^.

## COMMENTARY

Lost Mary states on its website that their products shall not be marketed in ways that appeal to youth, including avoiding ‘designs that are attractive to young people’ and ensuring that human models used in marketing materials ‘appear to be at least 25 years of age’^7^ – most likely as part of self-regulatory efforts. However, findings from the 2023 National Youth Tobacco Survey showed that Lost Mary was a commonly used brand among US youth, with an estimated 50,000 teens reporting use of the brand^8^. This was likely an underestimate, as Lost Mary was not one of the prespecified response options in the survey but was the most commonly reported brand in the open-ended ‘some other brand(s) not listed here’ option.

**Figure 1:**
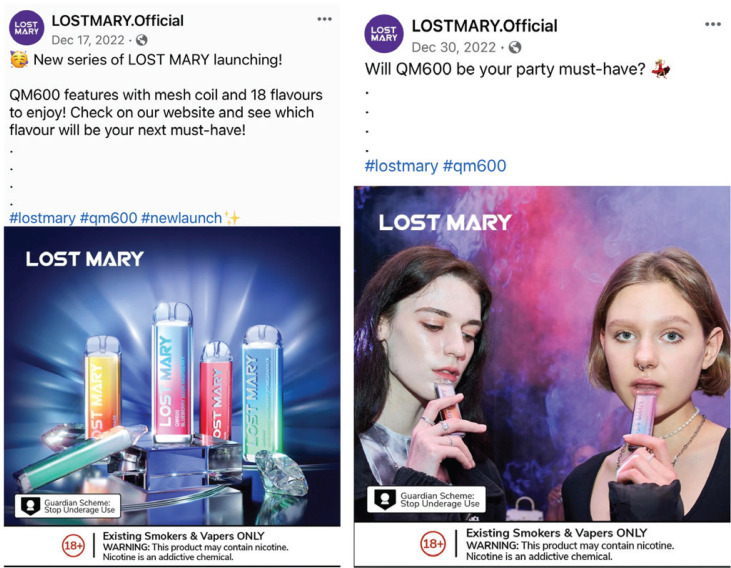
Example of ads collected as part of the coded sample, from the official Lost Mary Facebook page (www.facebook.com/LOSTMARY.Official.Global) posted in December 2022, with youth-appealing features including vibrant colors, flavor descriptors, young models, and emojis

To explore whether this youth popularity surge coincided with explicit marketing efforts, we conducted a content analysis of Lost Mary social media posts published between October 2022 and December 2023 on their official global brand Facebook, Instagram, and YouTube pages (see Figure 1 for example of ads). Manual and automated data collection techniques were implemented to capture postings due to variations in platform structure and material quantity and format. Automated collections of postings from Instagram and Facebook were conducted using Selenium WebDriver 4.0 for Chrome. WebDriver is a programming interface that allows users to automate web browsing behaviors. For this study, WebDriver was utilized to access the Lost Mary accounts and screen capture each posting during the specified timeframe. Manual data collection was conducted for YouTube uploads, where a single research member accessed the Lost Mary YouTube channel and collected postings for analysis. Of the 62 posts initially compiled, 9 Instagram posts were removed from the sample, as they were duplicates of posts found on Facebook, resulting in a final sample of 53 posts. We developed a codebook for various marketing features, including those unique to the platforms (e.g. video). The codebook was refined by test coding a sample of posts not included in the final sample. Content coding included the entire post, both caption and images, and captured various features, such as hashtags and emojis used in the caption; warning labels referring to underage sales or nicotine content; product details such as flavor and product design; the presence and perceived age of human models; and themes present in the ad, like ease of purchase and tech innovations. Two trained coders double-coded all posts, resolving discrepancies through discussion; inter-rater reliability was good (κ=0.88; 95% CI: 0.86–0.89).

We found that more than a third of the posts (35.9%) featured a flavor descriptor, and of these, most referred to fruits (84.2%) and/or ice (61.1%). This is concerning given that fruit flavors are still the most popular among current youth e-cigarette users (63.4%)^8^, followed by flavors including the word ‘ice/iced’ (57.9%)^8^. About two-thirds of the posts featured vibrant colors (67.9%), described in the content analysis guide as bright, eye-catching color palettes in contrast to images that were greyscale or muted in tone. Color, which the tobacco industry has consistently used to convey flavor^9^, is a common feature of e-cigarette advertising^10^ and has been found to appeal to youth^11-13^. As such, vibrant colors used in these posts may lead to positive taste and product expectancies, bolstering the effects of ads with flavor descriptors and/or substituting for their absence in ads without such descriptors. Although only 11.3% of the posts included images of people, they all featured models who appeared to be young adults (i.e. looked to be older than 18 and younger than 35 years), which has been shown to be attractive to young people^11,14,15^. The majority of posts (62.3%) also included emojis, a feature known to attract and engage youth^16^.

## CONCLUSION

The findings of this study suggest that Lost Mary may try to appeal to a younger demographic on global social media platforms. As part of the FDA’s continued efforts to address e-cigarettes that appeal to youth in the US (including flavored disposable vapes that are not authorized to be in the US marketplace), as well as calls for youth e-cigarette prevention globally^17^, our findings suggest the need for increased monitoring and regulation of youth-appealing marketing of these products, including on social media.

## Data Availability

The data supporting this research are available from the authors on reasonable request.
